# Supplementary material for: The Evolution and Characterization of the RNA Interference Pathways in Lophotrochozoa
Source: Genome Biol Evol. 2024 May 7;16(5):evae098. doi: 10.1093/gbe/evae098 (PMC11114477; doi:10.1093/gbe/evae098)
Supplement: evae098_Supplementary_Data [file evae098_supplementary_data.zip › Supplementary_materials.zip!/selected_miRNAs/NoGe_selected_miRNAs/mirdeep_pdf_files/NMRB01000004.1_Notospermus_geniculatus_strain_Ushimado_scaffold4,_whole_genome_shotgun_sequence_142.pdf]

Provisional ID	:	NMRB01000004.1_Notospermus_geniculatus_strain_Ushimado_scaffold4,_whole_genome_shotgun_sequence_142
Score total	:	116.4
Score for star read(s)	:	3.9
Score for read counts	:	111.3
Score for mfe	:	1.8
Score for randfold	:	0
Score for cons. seed	:	-0.6
Total read count	:	230
Mature read count	:	123
Loop read count	:	0
Star read count	:	107

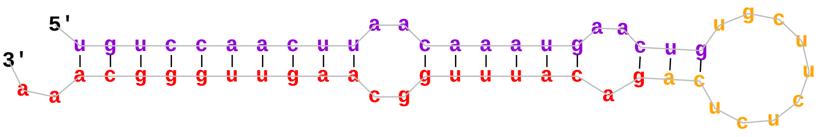

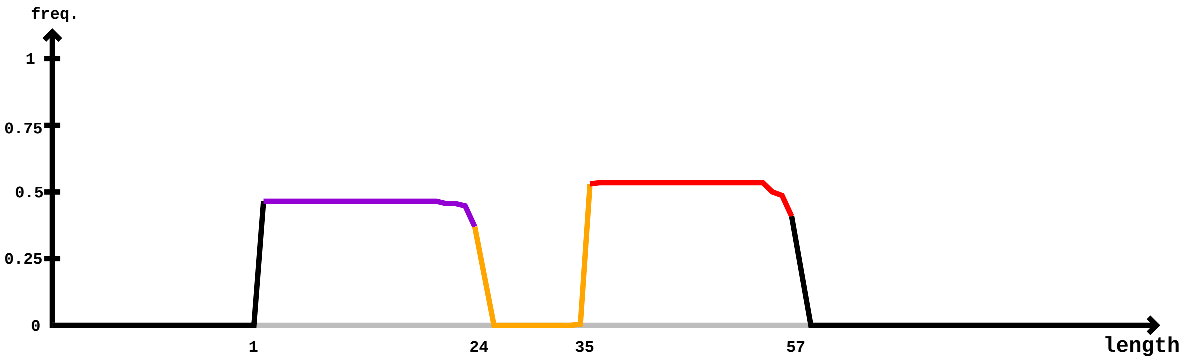

Star **Mature**

```
5'- ggugacuuggcuggagcaguuguccaacuuaacaaaugaacugugcuucucucagacauuuggcaaguugggcaaaauguuucagacaucaaauauuggcaaccaauauuggu  -3'   obs
    ggugacuuggcuggagcaguuguccaacuuaacaaaugaacugugcuucucucagacauuuggcaaguugggcaaaauguuucagacaucaaauauuggcaaccaauauuggu         exp
    ..(((..((.((((((((.((((((((((((..((((((..(((.........))).))))))..))))))))))))..)))))))).)))))(((((((...))))))))...  reads   mm   sample
    ....................uguccaacuuaacaaaugaacug......................................................................  18      0    Mm1
    ......................................................gacauuuggcaaguugggcaaa.....................................  6       0    Mm1

    ....................uguccaacuuaacaaauga..........................................................................  1       0    Mm2
    ....................uguccaacuuaacaaaugaac........................................................................  1       0    Mm2
    ....................uguccaacuuaacaaaGgaacu.......................................................................  1       1    Mm2
    ....................uguccaacuuaacaaaugaacu.......................................................................  9       0    Mm2
    ....................uguccaacuuaacaaaugaacug......................................................................  29      0    Mm2
    ....................uguccaacuuaacaaaugaacugu.....................................................................  3       0    Mm2
    ......................................................gacauuuggcaaguugggc........................................  1       0    Mm2
    ......................................................gacauuuggcaaguugggcaaa.....................................  3       0    Mm2
    ......................................................gacauuuggcaaguugggcaaaG....................................  1       1    Mm2
    ......................................................gacauuuggcaaguugggcaaaU....................................  1       1    Mm2
    ......................................................gacauuuggcaaguugggcaaaa....................................  2       0    Mm2

    ....................uguccaacuuaacaaaugaacug......................................................................  4       0    Fm1

    ....................uguccaacuuaacaaaugaacugu.....................................................................  2       0    Fm2
    ......................................................gacauuuggcaaguugggcaaa.....................................  3       0    Fm2

    ....................uguccaacuuaacaaauga..........................................................................  1       0    Mm3
    ....................uguccaacuuaacaaaugaac........................................................................  1       0    Mm3
    ....................uguccaacuuaacaaaugaacu.......................................................................  1       0    Mm3
    ....................uguccaacuuGacaaaugaacugu.....................................................................  1       1    Mm3
    ....................uguccaacuuaacaaauCaacugu.....................................................................  1       1    Mm3
    ....................uguccaacuuaacaaaugaacugu.....................................................................  1       0    Mm3
    .....................................................agacauuuggcaaguugggc........................................  1       0    Mm3
    ......................................................gacauuuggcaaguugggcaaa.....................................  1       0    Mm3

    ....................uguccaacuuaacaaaugaacu.......................................................................  7       0    Fm3
    ....................uguccaacuuaacaaaugaacug......................................................................  26      0    Fm3
    ......................................................gacauuuggcaaguugggc........................................  6       0    Fm3
    ......................................................gacauuuggcaaguugggca.......................................  3       0    Fm3
```

```
                    Star                             Mature
ggugacuuggcuggagcaguuguccaacuuaacaaaugaacugugcuucucucagacauuuggcaaguugggcaaaauguuucagacaucaaauauuggcaaccaauauuggu
......................................................gacauuuggcaaguugggcaa......................................   16   0   Fm3
......................................................gacauuuggcGaguugggcaa......................................   1    1   Fm3
......................................................gacauuuggcaaguugggcaaG.....................................   1    1   Fm3
......................................................gacauuuggcaaguugggcaaa.....................................   50   0   Fm3
......................................................gacauuuggcaaguuggCcaaaa....................................   1    1   Fm3
......................................................gacauuuggcaaguugggcaaaa....................................   22   0   Fm3
......................................................gacauuuggcaaguugggcaaaG....................................   3    1   Fm3
.......................................................acauuuggcaaguugggcaa......................................   1    0   Fm3
```
